# Drivers of psychological distress among first year female public university students in South Africa: A qualitative exploratory study

**DOI:** 10.1371/journal.pmen.0000566

**Published:** 2026-04-02

**Authors:** Mercilene Tanyaradzwa Machisa, Pinky Mahlangu, Yandisa Sikweyiya, Rachel Jewkes, Elizabeth Dartnall, Sinegugu Duma, Nelisiwe Khuzwayo, Managa Pillay, Millicent Maoto, Carrie Brooke-Sumner

**Affiliations:** 1 . Gender and Health Research Unit, South African Medical Research Council, Pretoria, Gauteng, South Africa; 2 Faculty of Health Sciences, School of Public Health, University of Witwatersrand, Johannesburg, South Africa; 3 School of Nursing and Public Health, College of Health Sciences, Howard College Campus, University of Kwazulu-Natal, Durban, South Africa; 4 Faculty of Health Sciences, School of Health Systems and Public Health, University of Pretoria, Pretoria, South Africa; 5 Office of the Executive Scientist, South African Medical Research Council, Pretoria, Gauteng, South Africa; 6 Sexual Violence Research Initiative, Pretoria, Gauteng, South Africa; 7 Oprah Winfrey Leadership Academy for Girls, Meyerton, Gauteng, South Africa; 8 Mental Health, Alcohol, Substance Use and Tobacco Research Unit, South African Medical Research Council, Parow, Cape Town, South Africa; Ss Cyril and Methodius University in Skopje: Saints Cyril and Methodius University in Skopje, NORTH MACEDONIA

## Abstract

Existing literature, primarily from high-income countries, highlights the first year of higher education as a challenging period for students. Academic demands, family expectations, social pressures, and relational difficulties are commonly associated with increased psychological distress during this time. In South Africa, research is mostly quantitative, lacking qualitative insights into the nuanced drivers of distress among first-year students, particularly young, female, and Black-African students. We aimed to explore drivers of psychological distress among Black-African first-year female students in South African universities and examine how they collectively influenced their mental health challenges. Fifty-four Black-African, female students aged 18–30years enrolled at three South African university campuses participated in three focus group discussions led by female researchers. The FGDs explored first-year students’ drivers and experiences of psychological distress while navigating the transition to university and their considerations around utilizing support services. We found that individual, peer, family, relationship, and institutional factors collectively contributed to psychological distress among students. Academic challenges including increased workloads and unfamiliar study methods, combined with struggle to secure residences, and social pressures to conform to peer lifestyles, exacerbated their distress. Financial distress, often intensified by delayed funding and family obligations like “black tax”, forced difficult choices and prioritisations. Additionally, controlling and emotionally abusive relationships, alongside partner infidelity, further undermined the students’ well-being. These interconnected stressors negatively impacted psychological distress which was aggravated by limited access to support services during the critical university transition. Findings suggest that first-year Black-African female students, especially those from low-income households, often experience psychological distress from intersecting academic, social, financial, and relational challenges. To improve wellbeing and academic success, universities must prioritise removing barriers to comprehensive support for first year students. Key actions include ensuring timely financial aid, expanding access to mental health services, academic orientation programmes, and implement initiatives that promote mental health, women’s empowerment and healthy relationships.

## Introduction

Psychological distress refers to a range of overwhelming mental and physical symptoms that many individuals experience as part of normal mood fluctuations, including feelings of sadness, anxiety, and bodily discomfort [1]. Although these symptoms are often temporary, psychological distress can sometimes compromise well-being and functioning thus indicating the early development of more serious mental health conditions such as major depressive disorder or anxiety disorders [1]. Many self-report instruments designed to measure depression and anxiety focus on capturing the presence and intensity of psychological distress symptoms, reflecting its central role in mental health assessment [1]. Psychological distress is especially important among young people due to its high prevalence and significant impact on their development, as it represents heightened vulnerability to mental health challenges during a critical transitionary life period [2–6]. Adolescents and young adults frequently encounter emotional challenges that can disrupt academic performance, social relationships, and overall wellbeing [7–9]. Elevated psychological distress in this group is also associated with increased risks of social withdrawal, loneliness, and in severe cases, suicidal behavior [4–6,10,11].

Within educational settings, understanding the dynamics of psychological distress is essential because these are primary environments where young people spend much of their time. Early recognition and intervention can help mitigate the adverse effects of distress on learning outcomes, social development, and mental health. Furthermore, educational institutions are uniquely positioned to implement preventive strategies and create supportive environments that promote psychological wellbeing among students [5,9].

### Drivers of psychological distress among university and college students

#### Transition from high school to university.

Being in the first year of university or college can be stressful and can lead to the onset of, or worsen existing, mental health problems for many young people [8,12,13]. For many young people, leaving home for the first time involves significant personal and developmental adjustments - such as learning to live independently, navigating academic demands, and forming new social networks [9,14]. Adjusting to university or college life also requires coping with the combined pressures of academic and social transitions, alongside the everyday demands of meeting personal needs [13,15]. During this transition, first-year students face the pressures of emerging adulthood, including making independent decisions and establishing a personal identity [16]. Difficulty adjusting to new freedoms and choices and worries about life and academic pressures, can increase psychological distress [13]. This, in turn, can contribute to higher rates of mental health problems, particularly anxiety and depression, that often are compounded by maladaptive coping mechanisms, especially alcohol and substance abuse amongst students [9,13]. Mental health problems pose significant risks to first-year students’ academic performance, social adjustment, physical health, and overall well-being [8,12,14]. Academic stress can also lead to social withdrawal, making it difficult for students to connect with peers and seek support. This isolation can exacerbate feelings of academic inadequacy, creating a vicious cycle where anxiety and stress both negatively impact social and academic performance [13].

#### Socio-demographic factors.

Socio-demographic factors linked to difficulties in adjusting to university or college life and increased stress levels include being a first-generation college student, a woman, a sexual minority, or from a lower socioeconomic background [12,17]. In Sub-Saharan Africa, factors such as poverty, food insecurity, gender and social inequality, and multiple chronic traumas may uniquely contribute to mental health challenges faced by first year students [7,8,13,18]. A recent national survey conducted across 17 universities in South Africa identified groups at higher risk of common mental disorders including younger, first-generation, female, LGBTQ, and Black students [7]. Students from lower socioeconomic backgrounds often face heightened psychological distress. Limited family financial support for basic living expenses, coupled with the burden of tuition fees, can add extra stress. Many are compelled to take on part-time jobs to cover costs which can further strain their wellbeing and capacity to focus on their studies [7,17]. Students facing such pressures may develop lower self-esteem and lack financial and emotional resources to participate in extracurricular activities that help build social connections and capital [12].

#### Family-level factors.

Family backgrounds can affect students’ experiences of psychological distress. Family support can alleviate psychological stress faced by students transitioning to university life, but it is not always available [12,19]. Studies mostly from high income country settings have found that many first-generation college students have a harder experience in adjusting, because they receive less emotional and financial support and guidance from their parents compared to students whose families have a history of college [20,21]. In the high income country settings, first-generation students often struggle to navigate academic support services like tutoring services, academic advisory support, and library resources [17]. They may feel overwhelmed and unsure about where to get help [21]. First-generation students were found to often face additional challenges from family expectations about their education while managing university or college demands [19]. In contrast, students with family members who attended university usually had an advantage. Their families offered emotional support, practical advice, and guidance about academic life. This support helped them adjust better and possibly led to greater academic success [17,21]. First-generation students often rely more on peers for support because they get less help from family. However, these peer networks can be unreliable, especially during the early transition to college when friendships are still forming. Students who build strong, supportive friendships usually experience less stress. Those who struggle socially or face discrimination based on social class or gender often get less emotional and informational support. This can increase feelings of isolation and distress [12,17]. However, it is still unclear whether the challenges faced by first-generation and continuing-generation students in South Africa are similar to those reported in high-income countries. While South African research shows that first-generation students often struggle with financial issues, academic under-preparedness, and social adjustment, there is limited data comparing their experiences directly with those of students in other contexts [22–24].

#### Gender-related factors.

Previous research shows that female college students face unique gender-specific factors that increase their risk for psychological distress and mental health problems. Key stressors include challenges in romantic relationships and friendships, which significantly affect their well-being [13,17]. Experiences of sexual violence including childhood trauma, intimate partner violence, or non-partner sexual assault, are strongly linked to higher levels of distress and more severe mental health issues [25,26]. In addition, difficulties with social integration and pressure to gain social acceptance and status at college add to their psychological burden [13,17] Socio-economic factors like food insecurity further worsen these problems, negatively impacting both mental health and academic success [9,13,17,25,26]. Female students identifying as lesbian or bisexual face significant mental health challenges, including high rates of depression, anxiety, substance use, self-harm, and suicidal ideation [27]. These issues are closely linked to experiences of family rejection due to sexual orientation, societal and cultural pressures on women, and religious stigmatization of minority sexual identities. Navigating their sexual identity in unaccepting environments often leads to self-stigma, emotional exhaustion, and increased vulnerability to homophobic abuse and discrimination, which further intensify psychological distress [27,28]. The vulnerabilities of female students to psychological distress and mental health challenges are shaped by the complex interplay of their intersectional identities including gender, race, and socioeconomic status and overlapping social determinants and drivers, which together compound and intensify their mental health risks and outcomes [11].

South Africa faces persistently high rates of teenage pregnancy, with nearly one in four girls becoming pregnant before age 20 [29]. Among female university students who are young mothers, the challenges of balancing academic demands, early motherhood, and financial strain are intensified by limited social support and absent fathers [30,31]. While child support grants provide some relief for basic child needs, they often bring additional financial expectations from extended family, exacerbating stress [30,31]. These intersecting factors contribute significantly to elevated mental health risks in this vulnerable population.

## Current study

Overall, the literature we reviewed suggests that different academic, family, social, and relational pressures contribute to a heightened risk of psychological distress and mental health issues such as anxiety and depression amongst students during the critical transition to higher education. However, to our knowledge most research on this topic in South Africa is quantitative, leaving a gap in understanding the nuances of the drivers of psychological distress among university and college students. There are few qualitative accounts of the drivers of psychological distress among first-year students in South African universities, particularly from young, female, and Black students and these have primarily focused on social and academic adjustment. This study further explores the experiences of Black African first-year female students of psychological distress and mental health challenges, and the academic, financial, social, and help seeking considerations that shape these experiences. By examining the complex interplay of individual, peer, family, intimate relationship, and institutional factors, the research sought to inform the development of a conceptual framework to guide future context-specific and tailored interventions addressing the unique mental health needs of university students in South Africa.

## Materials and methods

### Study design, settings & participant criteria

We conducted an exploratory qualitative study using focus group discussions (FGDs) to explore the drivers of psychological distress among female, and Black students ages 18–30 years, across three university campuses in South Africa.

### Ethics statement

Ethical approval for this study was obtained from both the South African Medical Research Council Human Research Ethics Committee (EC003-2/2022) and the University’s Biomedical Research Ethics Committee (BREC/00004253/2022). Permission to conduct research with students was granted by the University registrar, acting as the gatekeeper. Participants provided voluntary, informed, and written consent after receiving a thorough explanation of the study and having sufficient opportunity to ask questions. Participants provided voluntary written informed consent and agreed to being audio recorded as part of the FGDs. All FGDs were conducted in full compliance with the guidelines and regulations set forth by the approving Ethics Committees. During the FGDs, the experienced researchers monitored participants for distress and provided immediate support and referrals to appropriate services on campus and outside of campus as required. Participants received snacks and an R50 reimbursement for participating in the FGD.

### Recruitment

The study used purposive sampling to recruit participants. Various strategies were employed to recruit the participants, including posting flyers about the study in public areas around the campuses, sharing information on official institutional social media pages, and circulating invitations in student WhatsApp groups. The flyers outlined the inclusion criteria, study purpose, and the contact details of the study principal investigators. Inclusion criteria included being enrolled as a first-year undergraduate student and assigned female at birth, having interest in participation and giving written consent. Female students enrolled at other levels were excluded. Interested students were asked to express their interest by contacting the researchers on the phone number provided. Some students were invited to participate by a peer research assistant, and others were recruited through staff working in student support services, peer education, and health promotion.

### Data collection

Three focus group discussions (FGDs) were conducted with 54 first-year female Black-African students between April and May 2023. Each FGD involved a group of 15–20 participants. FGDs were facilitated by two female Black-African researchers (MTM and PM) and participants spoke in either isiZulu or English based on their preference. Each FGD lasted between 60–90 minutes enabling the voices of all participants to emerge and was guided by a semi-structured FGD guide (S1 Data**).** The FGDs aimed to explore the experiences, context, and drivers of mental ill-health among first-year female students. Participants were asked to reflect on the challenges they faced during their transition to university life, including time management, academic workload, financial difficulties, and dating relationships. The FGDs sought to understand the sources of frustration, stress, and mental health difficulties experienced by these students, as well as their coping strategies and behaviours. Probing during the focus group discussions continued until data saturation was achieved, indicated by the point at which no new themes or information emerged from the participants. The researchers facilitating the FGDs emphasized the importance of group perspectives and encouraged participants to share their thoughts collectively rather than focusing on individual anecdotes. While personal stories were welcomed, participants were reminded of the limits to privacy and encouraged to share only those experiences they felt comfortable discussing openly. The facilitators actively listened to the participants’ views and probed further to gain a deeper understanding of their insights and experiences.

### Data analysis

The audio recordings from the FGDs were transcribed verbatim, with IsiZulu sections translated into English. We employed an inductive thematic analysis approach to allow themes to emerge directly from the data rather than being constrained by a predefined framework [32]. The coding team comprising MTM, PM, CBS and YS immersed themselves in the transcripts through repeated readings to develop a deep familiarity with the data. Initial coding was conducted manually using MS Word, generating codes that were grounded in the data and reflective of the research questions and objectives. Instead of using a fixed codebook from the start, the codebook was developed step-by-step. As new ideas and patterns appeared during independent coding, they were added to the codebook to keep it flexible and complete. Then, the team worked together to group similar codes into themes and sub-themes, discussing how they related to each other. This back-and-forth process between the data and analysis made sure all-important insights, both expected and new, were included. This way, the analysis stayed truly inductive, allowing new codes from the data to shape the codebook rather than being left out if they weren’t in the original codebook. Member checking was conducted with students in each campus who provided feedback on the findings. This involved sharing and validating illustrative quotes drawn from transcripts.

### Reflexivity and positionality

Throughout the research process, the FGD facilitators (MTM and PM) engaged in ongoing reflexivity to remain aware of and mitigate the influence of their personal experiences and potential biases on data gathering and interpretation. They continually reflected on their identities as senior Black African women and seasoned academics, considering how these positionalities might have shaped participant engagement and the overall group dynamics. Reflective field notes and debriefing sessions were used to capture insights about group dynamics, participant interactions and observed emotional reactions during FGDs. During the analytical phase, the research team (MTM, PM, CBS, and YS) debriefed how their varied lived experiences and backgrounds could affect theme identification and coding decisions. This reflexive process strengthened the credibility of the findings by ensuring that interpretation of data remained closely aligned with participants lived experiences rather than researchers’ assumptions.

## Results

Overall, findings suggest that complex, interconnected drivers of psychological distress undermine both the academic performance and well-being of Black African first-year female students in public South African university campuses. Four main themes emerged from the data as domains of drivers of psychological stress. Students reported on domains of psychological distress driven by their experiences of navigating academic transition, social adjustment, dealing with financial pressure and barriers to utilizing available services. For each domain we drew subthemes that described how their experiences of psychological distress in the four domains were related to how they perceived themselves (individual) and how they related to peers, intimate partners, families and navigated the institutional environment. The findings demonstrate how their experiences in different domains overlapped and interacted to intensify psychological distress and collectively affect the mental health and academic success of this vulnerable group. There was low engagement with campus support systems due to lack of awareness, stigma, accessibility challenges, and perceived inadequacies in service provision. Such service underutilization further aggravated the complex interplay of academic, social, financial, and institutional stressors that collectively undermined students’ mental health and academic success (S2 Data**).**

### Academic adjustment

Participants reported significant challenges in transitioning to the academic demands of university. They observed that many first-year students were overwhelmed by the increased workload, the complexity of coursework, and unfamiliar study strategies:

*“One of the things that make first years have problems, at first is that we try to adjust to schoolwork. It’s now big. It’s getting out of hand. There are now practicals, and you need to go to campus, and you need to do four modules compared to high school. You used to do eight subjects, but a lot has changed, and you need to adjust to new ways of studying because the way we used to study is no longer the same as what we are doing right now”* FGD2, Campus 2 Participant

The transition from the supportive learning environment of high school to the more independent approach required at university often led to feelings of confusion, self-doubt, and inadequacy. Many students also found the teaching styles of university lecturers to be markedly different from their high school experience, where they felt there was greater support for learning.

A participant shared their experience:

*“…I never did accounting in high school, and there are many challenges. You have to learn, and we are not familiar with lecturing. You are used to being taught ‘this, and then that’ but now you are being lectured, and you don’t understand anything. You don’t even know the basics. You see that you are getting lost. You just feel stupid. See, I am stupid.” –* FGD2, Campus 2 Participant

Other challenges encountered with adapting to the university environment, included the perceived strictness of lecturers and expressed frustration with group work assignments, particularly when peers did not contribute equitably. “Ghost group members” who did not participate in the work, increased workload for other group members. Missed submission deadlines, and a lack of flexibility from lecturers regarding extensions, further exacerbated these difficulties. A participant highlighted the stark contrast between the university and high school environments,

*“Here in varsity if you don’t submit your assignment on time then the system locks you out. And it’s very rare for a lecturer to be lenient and extend the due date for an assignment. Sometimes for group work, it possible to get selected in a group with “ghost members” (lazy members who disappear and do not contribute to group work). [Group Laughing]”* FGD3, Campus 3 Participant

Some participants expressed a lack of awareness and difficulty in accessing academic support resources. They often struggled to locate lecturers’ offices and were unsure about the process of scheduling appointments, which hindered their ability to seek assistance:

*“The problem is that we don’t know where the offices for the lecturers are located, and some lecturers want you to set an appointment before coming to their offices. It’s a long process. We are not yet familiar with a lot of buildings on campus, and we only know a few offices where we can find our lecturers. Having no idea where we can find most of our lecturers is a big issue.”* FGD3, Campus 3 Participant

Several participants expressed anxiety about the possibility of failing tests and receiving poor grades. They attributed their concerns to insufficient study time and the need to cover a large volume of material before assessments. They emphasized that failing tests could negatively impact their funding for the following year. One participant described their experience as traumatic:

*“I completed a certain module a week before we closed. I didn’t know that was my last test and we are writing on campus. Like everything got compacted into two tests. Like, how can you do that? How am I going to cover all these topics? And you are supposed to get As. You are supposed to get As to maintain the bursary. I mean okay at least there is like counselling here on campus, but yoh, I was left traumatised”.* FGD2, Campus 2 Participant

Other participants expressed concerns about disappointing their families, who often held high expectations for their academic success. Some criticized their parents for not fully understanding the academic pressures they faced and for not providing emotional support when it was most needed, leading some students to resort to negative coping strategies:

*Parents also put us under a lot of pressure regarding our schoolwork and provide less support to us. There is a student that I know, she was stressed about school and her parents didn’t take her serious until she attempted to commit suicide.* FGD1, Campus 1 Participant

A significant challenge identified among first-year female students was the difficulty in securing university residence, which contributed to their psychological distress. Students from distant or rural areas faced greater barriers compared to those from places close to the university campuses. Unlike local students who could ask family for support, those from further away often found themselves isolated, without family networks to assist them. This exacerbated feelings of vulnerability and psychological distress, illustrating how geographic and social isolation intersected with the challenges of transitioning to university life. One participant explained:

“*You see, kids who come from places that are far face greater challenges. If you are from near or have family, you can at least phone and say, ‘Please help me.’ But if you are coming from far, you are stuck here at the university with nowhere to turn because you do not have family in the province.”* FGD2, Campus 2 Participant

The persistent nature of the residence problem emerged as a significant concern among participants, reflecting a long-standing issue that remains unaddressed by institutions. This exacerbates students’ psychological distress and undermines their academic stability and sense of belonging. As one participant noted,

“*Especially if you are coming from another province you might end up having to go back without getting residence which is disadvantageous and need to be taken care of. Unfortunately, with this issue of residences, I think institutions are not determined to fix residence challenges hence it been years experiencing the same issues.”* FGD3, Campus 3 Participant

### Financial distress

Many participants described financial challenges as a significant source of psychological stress for students in this setting, with most relying on the government-administered National Student Financial Aid Scheme (NSFAS) bursaries to cater to their basic needs. Participants explained that the expectation for first-generation female students from low-income households to provide financial support for their families emerged shortly after they began university studies, or when their families became aware of their financial aid:

***“****Yes, they are expecting. If you have NSFAS funding, they know you get a monthly allowance therefore expect money from you. Now I have more duties than I had before or different expectations like being able to provide things for home. Not saying provide things, but more things are being expected from me whereas I was not used to that transition.”* FGD2, Campus 2 Participant

Other participants said many first-year female students from low-income households experienced stress due to conflicting financial expectations from their families. They explained that despite receiving limited financial support, some female students felt pressured by their families to use their limited resources to support others at home. This added another layer of stress, as students felt burdened by both academic demands and family financial pressures.

*“Another thing which I think happens to most of us is that when we ask for some money from our parents, they reprimand and accuse us of being careless with the way we handle our finances in varsity. Hearing my parents complaining about money problems at home instead of assisting me financially increases my stress levels.”* FGD3, Campus 3 Participant

Participants said first-generation students from low-income households were burdened by their families’ expectations that they provide and contribute significant amounts of the funds they received to their family welfare. This phenomenon, often referred to as “black tax” emerged as a key concern for participants.

*“I think the other stressful thing is black tax. Sometimes, even though one will not agree, back home they expect her to give them R500 from the money she gets from NSFAS or bursary to help her siblings because some of them, you find she is the first generation that will graduate from her family. So, if the parent maybe doesn’t have a good job and then you have to make a plan”.* FGD2, Campus 2 Participant

Other participants expressed frustration with their families’ perceived sense of entitlement and manipulative tactics when requesting financial assistance. They explained that refusing to comply with these demands was often interpreted by their families as neglectful and uncaring behaviour towards those who had raised them. One participant explained that:

*“I don’t mean this in a bad way but some of our parents think that when we are here and getting NSFAS, it’s like we are getting a lot of money. They raised and sent us to school from nothing. They now think that we must help them because there is money that we are getting. So now when you are saying you cannot help your parents, they think that you have forgotten them back home.”* FGD2, Campus 2 Participant

Many participants described experiencing feelings of guilt when they were unable to send money home to their families, particularly when they were compared to peers who were financially supporting their loved ones. This guilt was often exacerbated by the knowledge of their families’ financial struggles, their manipulative devices and the pressure to meet societal expectations around “black tax”. Participants often felt compelled to provide financial assistance, even if it meant sacrificing their own needs, due to parental expectations and the fear of disappointing their families. As one participant described:

*“The minute you do not send money, you will feel guilty because you know the situation you left at home. Maybe you have to sacrifice some money because you will think why can’t I also do like others and give them money too.”* FGD2, Campus 2 Participant

Participants reported significant delays in receiving financial aid during their first year, as applications were only processed after full enrollment. This financial uncertainty caused considerable stress and anxiety, with some female students contemplating dropping out of university. One participant explained, “*Having no NSFAS funding causes a lot of stress. You will be overthinking about dropping out of school because having no funding causes a lot of stress.*” FGD3, Campus 3 Participant

This lack of financial support from families combined with delayed and insufficient university funding, further exacerbated their mental health challenges. As another participant noted, *If the university doesn’t have funding, then you must ask for everything from your parents. When other students have funding, this ends up affecting your mental health when they are happy that their money has come through and you do not have anything”.* FGD2, Campus 2 Participant

Other participants noted that female students with children often bear full financial responsibility for their children, especially when the fathers are absent and do not provide support. Some female students from low-income households receive child support grants from the South African Department of Social Development (DSD), which are intended to cover the child’s basic needs. However, when the child lives with the grandmother, the grant is typically allocated to the grandmother as the primary caregiver, not the student. Despite this, the grants often create additional expectations from extended family members for financial contributions. These dual financial pressures from both bursary funds and child grants compound the stress and psychological burden these students face. A participant explained, “*Another girl was also having kids at home… So, they call her every month end and ask her, “When are you sending money?”* FGD2, Campus 2 Participant

#### Challenges in managing finances*.*

Managing finances also emerged as a critical and difficult adjustment for first-year female students, contributing to ongoing psychological distress as they navigated unfamiliar responsibilities and competing demands. Participants described managing their personal finances as challenging as for the first time they were responsible for budgeting and prioritizing expenses. One participant captured this struggle, saying, “*You have to ‘parent’ yourself. That is where the problem starts. Having no one to provide you with guidance.”* FGD1, Campus 1 Participant. The difficulty of balancing limited funds was evident in their accounts of deciding between essentials like food and clothing: “*Sometimes you have to sacrifice food to buy clothes,”* and “*When you are doing your own grocery, the price of every item matters the most.”* FGD1, Campus 1 Participant. This shift from a family-supported environment to independent financial management resulted in first year female students’ distress and uncertainty.

Financial pressure was especially acute around the time allowances were disbursed. Participants reported that the arrival of their NSFAS funds triggered anxiety about stretching limited resources across the entire month. One participant explained, “*Once the allowance starts reflecting on our accounts, we begin to become stressed about how we are going to use R1500 (USD 80) to sustain us throughout the month.”* FGD3, Campus 3 Participant. Peer pressure to spend on social activities further complicated budgeting decisions, creating internal conflict between the desire to fit in and the need to prioritize essentials. As another participant noted, “*Seeing other students going out to celebrate receiving their allowances makes me more stressed... I feel pressured to go out too, though I know I must use that money wisely.”* FGD3, Campus 3 Participant

#### Financial stress and transactional relationships.

Participants described the strategies they use to cope with intense socioeconomic pressures, explaining that engaging in relationships with “blessers” who are older, wealthier men who provide financial support, is often driven by the urgent need to support families, escape poverty, and meet social expectations, rather than by simple personal choice. They highlighted how these pressures contributed to psychological distress, as they navigated stigma, judgment, and the burden of maintaining certain standards in appearance while managing financial insecurity. They called for empathy and understanding of the layered motivations behind such actions, and the mental health challenges intertwined with these survival strategies.

*“If ever there is someone you know who is dating a blesser or doing whatsoever, please do not judge those people. Try to understand why they did these things in the first place because you find that some of them are expected to support their families back home. One may have left a home that does not have anything at all and then she will have the pressure of trying to maintain standards. It’s a lot”.* FGD2, Campus 2 Participant

The participants highlighted the tension between meeting academic responsibilities and fulfilling the expectations of their benefactors, noting that failure to attend to the blesser’s needs often results in losing their financial support. The pressure to maintain this balance contributes to heightened psychological distress and anxiety, driven by socioeconomic challenges and the use of transactional relationships as a coping strategy. One participant explained:

“*Maybe you have found yourself a blesser. Now you have to balance when the blesser needs you, you have to be there and your books are there too and you must pass, because when you don’t go to the blesser, then you will not get anything. You won’t get money; you won’t get anything if he doesn’t get you.”* FGD2, Campus 2 Participant

### Social adjustment

Participants described pressure to keep up with lifestyle trends and material possessions as a key reason some female students date older, wealthier men. Many reported that their financial resources were insufficient to cover campus expenses, especially for those from disadvantaged backgrounds who often had additional responsibilities like supporting themselves or their families. Despite limited funds, students felt compelled to match their peers’ spending habits, even if it meant exceeding their budgets. One participant explained that differing standards of living in university create feelings of misunderstanding and isolation for less privileged students when friends expect them to join in activities or purchases:

*“When going with friends they expect you to go and if you tell them, you don’t not have money, they will not understand hence they do not relate to an unfortunate background. Sometimes we often feel pressured to buy unnecessary things that exceed our budget if we notice that other students are buying a lot of things”.* FGD3, Campus 3 Participant

Participants reported experiencing significant psychological distress linked to social pressures and constant comparison with peers, particularly regarding financial status and appearance. The challenges of sharing residences heightened their awareness of these disparities, intensifying feelings of inadequacy. Many felt compelled to maintain a certain image to fit in and avoid negative judgments. This pressure was especially acute for those lacking additional sources of income, sometimes pushing them toward risky behaviours to generate extra money. As participants described:

***P:*** *“Firstly, one needs to dress nice… and doing shopping requires a lot of money. One has to dress nice without repeating clothes to appear attractive to others.” [Group laughing]*

***P:*** *“One might make silly comments if you are always wearing the same hat every day.”*

***P:*** *“And that might make you feel even ashamed to ride a school bus.”* FGD1, Campus 1 Participants

Participants also described how allocations for university residences often placed students in shared living environments with peers from diverse socioeconomic backgrounds, which created significant psychological distress and social pressure. Students living with roommates from financially stable families felt compelled to maintain certain standards they could not afford, leading to feelings of inadequacy and vulnerability. This environment sometimes introduced them to risky behaviors to generate extra income, driven by the desire to fit in or keep up appearances. One participant described this pressure, stating, “*Sometimes we stay with postgraduates and some of us are trying to reach the standards and then end up being introduced to some certain situations whereby you have to do stuff to... well to maintain that standard that you cannot reach. Then at some point you end up regretting and getting into stuff that you never thought you will ever be in sometimes.”* FGD2, Campus 2 Participant. Another reflected on the influence of financially stable roommates, saying, *“Maybe you might sometime stay with a roommate who is, maybe her background, comes from a family that is financially stable and then at some point she will do stuff that will make you have an interest in them... Then you start learning new ways of making extra income.”* FGD2, Campus 2 Participant

The pressures related to appearance and dress codes among first-year female students had profound mental health implications, exacerbating feelings of insecurity, low self-esteem, and social anxiety. Constant comparison with peers who could afford fashionable clothing created a persistent sense of inadequacy and exclusion, which intensified psychological distress. This shows that students struggled to reconcile their financial limitations with the social expectations of university life. One participant described this experience: “*Sometimes the dress code creates pressure because some people on campus look at you in ways that make you feel uncomfortable or insecure. This leads you to try to dress like them, even when you cannot afford it, because the money you have only covers your basic needs.”* FGD2, Campus 2 Participant

### Navigating intimate relationship dynamics

First-year female students experienced psychological stress due to intimate relationship problems, including control by older male partners, male entitlement, cheating, and conflicting relationship expectations. These issues negatively affected their mental health and academic performance. A key concern raised was their involvement with older male students, many of whom displayed controlling and isolating behaviors. As one participant explained, “*Another thing that I want to add is that sometimes, isn’t when we arrive as first years, it is rare for you to date another first year. So, like when you are dating seniors, they are always calling you and they isolate you from your friends so that whatever he says to you, you will feel like it’s the right thing.*” FGD2, Campus 2 Participant. This control was identified as extending to constant monitoring and restrictions on social interactions, contributing to emotional abuse characterized by verbal aggression and gaslighting. Another participant noted, *“Sometimes guys like to dictate how their girlfriends should dress and whom they should hang out with. If a girl doesn’t follow those rules, then hell breaks loose.”* FGD1, Campus 1 Participant

The controlling nature of these relationships often prevented healthy communication, increasing first year female students’ stress and emotional exhaustion: “*Knowing that my boyfriend doesn’t trust me and keeps on dictating how I should live my life and whom to hang out with increases my stress level. The worst part is when you can’t even have a simple conversation with your boyfriend because of how he reacts to a lot of thing”.* FGD1, Campus 1 Participant. Some participants also discussed the emotional challenges that emerged in transactional relationships where male partners felt entitled to sex after providing financial support. One participant shared:

“*You find that other people get violated within relationships. A person will say, “No I have spent this money on you. Now when we must do this you are telling me what to do, it happened a long time ago. So why can’t you move on? Things like that.”* FGD2, Campus 2 Participant

Low commitment from male partners was also reported as causing significant psychological stress for female students, leading to feelings of hurt, betrayal, and emotional distress. One participant explained:

*“Males in varsity are never serious when it comes to romantic relationships, it’s like they plan to deliberately hurt girls. In my own experience, I’ve been in a couple of relationships ever since I arrived in varsity, but it was not a serious thing. What I noticed is that female students who are in serious relationships with comrades get hurt the most because they get played.”* FGD3, Campus 3 Participant

Participants also highlighted that some male partners dated multiple female students simultaneously, leading to feelings of betrayal and insecurity: *“Sometimes a guy may be dating three girls at once without hiding it. It’s worse for a girl who already has strong feelings for that cheating guy.”* FGD3, Campus 3 Participant. Another participant also spoke about non-cheating partners displaying obsessive and overprotective behaviors, which were emotionally draining: “*A guy that is not cheating on you becomes too much… he becomes overprotective of you and obsessed.”* FGD1, Campus 1 Participant

Balancing academic responsibilities with relationships demands further intensified female students’ psychological stress. Participants described partners, especially those not studying, who failed to understand academic pressures and expected constant availability:

*“Those with boyfriends that they cook for at residences. Some boys do not understand how busy you will get. You have told him how busy you will be, like “So you will not have time for me.” And other boyfriends who are not studying get cooked for here. A person will say, “What have you cooked there mama? If you are dating someone who is not studying when you are studying, you find that you have schoolwork, and he won’t understand that today I can’t show up because it’s a lot. Like I have to study. He expects you to be there when he wants you to be there. That’s just not it.”* FGD2, Campus 2 Participant

### Barriers to utilizing campus mental health support services

We found significant gaps between the availability of campus mental health services and students’ awareness, access, and satisfaction with these services. Although counselling and psychological support exist on campuses, many students reported difficulty in accessing them due to systemic and logistical barriers. Several participants described the booking and application processes as cumbersome and frustrating, often resulting in long waiting times or fully booked sessions. One student shared:

*“The support systems here at varsity I tried to apply, it kept on repeating the same thing to me. You can’t apply. You apply and they say it’s fully booked, fully booked and I ended up giving up.”* FGD2, Campus 2 Participant

Others highlighted a lack of clear information about where and how to access services, with some stating they were unaware of the location or contact details of counsellors. This lack of visibility and accessibility was a major deterrent:

**P**:*“We lack information about these services. If I were to start searching for these services, I’m sure that it would take me a lot of days to find them.”*

***P****: “Another thing is that we don’t utilize those services on campus because their entrances are hidden... it’s difficult to locate venues for psychologists. Maybe it would have been better if there were big boards with signs directing us to the location of the psychologists.”* FGD1, Campus 1 Participants

In terms of service quality, some students who attended counselling sessions expressed dissatisfaction with the lack of structured guidance. One participant explained: *“I once attended counselling here... but it didn’t feel structured. I was put in a space with too much freedom, where I was expected to come up with my own solutions without clear advice or options laid out. I needed someone to guide me through possible steps, not just listen broadly.”* FGD2, Campus 2 Participant

Participants also identified anticipated peer stigma as a significant barrier to seeking mental health support on campus. Mental health concerns were frequently minimized or mocked within peer groups, fostering an environment where attending counselling was stigmatized and ridiculed. Additionally, students cited heavy academic workloads and fear of negative judgment from friends as further deterrents to accessing services:

**P:** *“It’s time, we are always busy with schoolwork.*

***P:*** *The type of friends that we have also prevents us from seeking help from those services. So, to avoid being labeled as a ‘lunatic’…“* FGD1, Campus 1 Participants

Participants also described denial and fear of confronting personal issues as significant factors contributing to their underutilisation of campus mental health services. Many expressed reluctance to face difficult truths or take necessary steps to resolve problems, which hindered their engagement with therapeutic support. One participant explained, *“I think that one of the reasons we don’t attend counselling is because we fear dealing with the truth.”* FGD3, Campus 3 Participant. This sentiment was widely shared, with participants highlighting the discomfort of confronting realities such as ending a toxic relationship when advised by a psychologist as explained by another participant:

*“For example, if you talk to the psychologist about a toxic boyfriend then obviously the psychologist will advise you to dump your boyfriend which is something that one doesn’t want to admit. So, we really don’t want to face the truth.”*FGD3, Campus 3 Participant

## Discussion

This study contributes to the limited qualitative evidence of the diverse experience of female students in transitioning through first year in South African university settings. We found that academic adjustments, including increased workloads and unfamiliar study methods, overwhelmed students, leading to feelings of confusion and inadequacy. The transition from a supportive high school environment to a more independent university setting further exacerbated female students’ academic-related stress, as students often perceived lecturers as less approachable and face challenges like strict deadlines and group work dynamics. The pressure to maintain high academic performance, often tied to financial aid eligibility, created a fear of failure that contributed to emotional distress for some. Female students spoke about financial stress that was exacerbated by delayed government funding and limited family support, forcing students to prioritize their families’ needs over their own, resulting in feelings of guilt and inadequacy. This was particularly acute for first-generation students from low-income households who felt obligated to contribute financially to their families, a practice often referred to as “black tax.” Social adjustment meant dealing with the pressure to maintain a lifestyle comparable to peers and this often led to transactional sex and relationships as students found this an avenue to cope with financial burdens. Controlling and isolating behaviours exhibited by male partners were experienced as emotionally abusive and stressful, contributing to feelings of inadequacy and isolation. The infidelity and lack of commitment by male intimate partners further exacerbated emotional distress for those who were in relationships and some female students experienced feelings of betrayal and insecurity which negatively affected mental health. Difficulty with securing residences, with some having to stay outside of campus, away from social support exacerbated distress. The data showed that challenges experienced by female first year students were interconnected and impacted academic performance and a cycle of psychological distress among first-year female students.

Participants highlighted the challenges faced by students who are suddenly thrust into a world of independence without adequate guidance or support to cope with the more complex university learning. The findings also highlight the limitations of the educational system in preparing students for the realities of higher education and adulthood. In high schools, many students benefit from a highly structured environment with strict rules and considerable external support from teachers, parents, and the school system. However, when transitioning to higher education, this level of support significantly diminishes, requiring students to navigate the complexities of independent living and academic pursuits largely on their own. This shift can make the transition challenging, as students must adjust to greater autonomy while managing academic, social, and personal pressures without the same support they had in high school. Poor orientation and lack of preparation for the demands of university academics can contribute to the stress experienced by students as they transition into adulthood. Previous studies have also shed light on students’ attribution of their adjustment challenges to their heavy workload, lack of guidance and commitment from their lecturers [15,33].

Financial strains faced by many students were a key driver of psychological distress and that finding underscores the importance of addressing issues related to student financial aid, cost of living, and support services to ensure the well-being of students transitioning to higher education. Consistent with previous South African studies [34,35], this research found that financial stress is a significant source of anxiety among first-year students, particularly those reliant on government financial aid like the National Student Financial Aid Scheme (NSFAS). Studies have shown that financial difficulties can lead to higher stress levels than academic demands or social factors [35]. Delays in receiving financial aid can exacerbate worry about meeting basic needs and increase the risk of dropping out, negatively impacting psychological adjustment [34]. While financial stress can affect students at all stages, first-year students may be particularly vulnerable due to the transition to university life [36]. However, this study extends previous research by presenting additional dimensions that compound financial stress among first-year female students, including family demands, “black tax” and the desire to maintain a lifestyle comparable to peers. These factors contribute to the complex interplay of financial burden and personal needs, further highlighting the challenges faced by first-year female students.

The study findings reveal a complex interplay of power dynamics, gender stereotypes, and vulnerability within romantic relationships that significantly exacerbate the distress experienced by first-year female university students. Many participants reported experiencing controlling, isolating, and emotionally abusive behaviours from their male partners, which were often intensified by the pressures of academic life. Infidelity emerged as a prominent concern, contributing to heightened feelings of betrayal and insecurity among the students. Additionally, some participants described relationships with older, more experienced male students, which frequently involved feelings of isolation and pressure to conform to their partners’ expectations. These findings suggest that male partners were often unsupportive or dismissive of their girlfriends’ academic ambitions, further straining the relationships and negatively impacting the students’ emotional wellbeing. They are consistent with other studies that implicated gender inequitable relationship dynamics or experiencing partner abuse as drivers of female students’ mental ill health [7–9,25]. These findings also underscore the need for comprehensive education, gender transformative interventions and support for students to promote healthy relationship dynamics, consent, communication, and boundary setting.

### Study implications

To alleviate financial stress among first-year female students, South Africa’s post-school education sector must implement timely financial support, especially for low-income students, while addressing broader social inequalities. Building financial management and problem-solving skills is essential. Policymakers should recognize burdens like “black tax” and its mental health impact. Universities should provide counselling and financial literacy workshops, which improve financial knowledge, self-efficacy, and reduce stress, empowering students to manage finances better and alleviate financial distress [37,38]. Universities must address the issue of limited residence availability to reduce distress, particularly among first-year students from outside the province. Residence allocations should be completed prior to arrival, with a priority on accommodating as many female first-year students as possible on-campus, to ensure proximity to social networks and university support systems.

To effectively reduce psychological stress among first-year female students, interventions should enhance emotional coping, stress management, and problem-focused strategies tailored to their lived experiences. Evidence supports combining psychoeducation, mindfulness, self-care, and coping skills to mitigate anxiety and mood problems [12,39]. Small group participatory workshops can provide opportunities for peer support that can help stress [17]. However, there is a significant gap in contextually relevant, evidence-based mental health programs for South African students that address intersecting social vulnerabilities [40]. Prioritizing research on diverse coping and help-seeking behaviors is critical to developing culturally sensitive, targeted support that advances mental health equity and student success.

Most South African Universities also provide counselling services that help students navigate the increased demands of college coursework. This study and others have shown that student use of campus mental health services is limited by several factors, including lack of awareness about available support, negative perceptions of service providers, cultural beliefs, concerns about confidentiality, and skepticism regarding the benefits of accessing services [41].The findings align with broader evidence from South Africa, where mental health stigma is deeply rooted in cultural beliefs, misinformation, and social norms that discourage open dialogue and treatment seeking [42].The findings indicate the urgent need for culturally sensitive stigma reduction initiatives and accessible, non-judgmental mental health care tailored to the unique experiences of Black South African students. Furthermore, targeted efforts to improve campus support services visibility, cultural relevance, and trust are essential to enhance access to campus mental health services, particularly for those facing intersecting vulnerabilities.

Strengthening first-year orientation programmes to better facilitate students’ transition into higher education is critical. Orientation programmes have potential to raise awareness about mental health and counselling services, tailored academic support such as tutoring and study skills workshops, establishing peer support groups, and improving problem-solving capacities [12,17]. South African universities already offer academic support programmes addressing gaps in preparedness through tutoring, academic advising, and counselling services that help students manage the increased demands of university coursework [8,40,41].

Promoting gender equity requires addressing systemic issues and fostering a safe, supportive campus culture where students can report abuse without fear of retaliation. In line with the National Policy Framework on Gender-Based Violence in post-school education, universities must provide comprehensive psychosocial support for survivors, including counselling and safe spaces for healing [43,44] Empowerment and social norms change programs should raise awareness about sexual harassment, challenge victim-blaming, inform students about risks and reporting processes, and encourage reporting.

### Limitations

This study has several limitations that warrant careful consideration. The exclusive focus on public universities, predominantly enrolling Black African, low-income, and first-generation students, limits the generalizability of findings to first-year female students from other demographic backgrounds. Additionally, the voluntary participation of students may introduce self-selection bias, as those willing to engage might differ systematically from the broader population. While the facilitative focus group approach resulted in rich, collective dialogue reflecting common experiences, it remains uncertain whether these insights represent the full diversity of first-year female students’ realities or are specific to the groups studied. Another limitation of this study is the use of large focus groups, with sizes ranging from 15 to 20 participants, which exceeded commonly recommended numbers in qualitative research which can increase risk of confidentiality breaches and heightened peer pressure or social desirability bias. Larger groups were convened due to participant scheduling constraints, allowing for diverse perspectives in fewer sessions outside academic hours. To mitigate these risks, experienced facilitators emphasized confidentiality, clarified the voluntary nature of sharing sensitive information, and promoted individual responses and balanced participation to reduce the influence of dominant voices and peer pressure, consistent with methodological guidance on focus group saturation and group dynamics [45–47].These constraints highlight the critical need for further in-depth research incorporating more diverse and representative samples to deepen understanding of intersecting vulnerabilities affecting student mental health and academic success.

## Conclusions

This study highlights that many first-year female students at the South African campuses examined experience psychological distress arising from intersecting identities and the complex challenges of social, financial, and academic adjustment. These difficulties are further influenced by their relationships with peers, intimate partners, and families, as well as their awareness of and access to institutional support during the transition to higher education. The findings underscore the urgent need for evidence-based, contextually relevant mental health promotion interventions tailored to female students facing overlapping social vulnerabilities. Developing culturally sensitive, targeted support services is essential to effectively reduce psychological distress, promote wellbeing, and improve academic outcomes.

## Supporting information

S1 Data Semi-structured FGD guide. (DOCX)

S2 Data Framework illustrating drivers of psychological stress among black first-year female students from low-income households in a South African Public University. (DOCX)

S3 Data COREQ checklist. (DOCX)

S4 Data Codebook and minimal data. (DOCX)
