# Supplementary material for: Drivers of psychological distress among first year female public university students in South Africa: A qualitative exploratory study
Source: PLOS Ment Health. 2026 Apr 2;3(4):e0000566. doi: 10.1371/journal.pmen.0000566 (PMC13046107; doi:10.1371/journal.pmen.0000566)
Supplement: S1 Data — (DOCX) [file pmen.0000566.s001.docx]

**Excerpt of FGD guide**

**Introduction:**

Young women, including first-year female students, often face unique challenges that can significantly affect their mental health. Today, we want to open a discussion to share our experiences and observations as first-year female students.

**Mental Health Challenges:**

1. What do you think are some of the mental health challenges that first-year female students experience? As you discuss, please share examples and insights on what you believe are the main sources of frustrations and challenges.
   - Probes: Thinking about the change from high school and entering into varsity what are the other challenges that students face? How do these challenges affect mental health?

**Coping with Challenges**

1. Please share with us your views on how first year female students are coping with all the frustrations and mental health challenges they face? What are some of the ways female students help themselves to manage stress, frustrations and mental health challenges?

**Institutional and other support**

1. What are your thoughts on the mental health and well-being support provided by the university/college to first-year female students? Please share from your own experience or what you have heard about the support first year females receive from the university.

- Probes: What other support programmes do we know off that could help first year females cope with adjusting and dealing with university life and what are your views about them.
