# Supplementary material for: Drivers of psychological distress among first year female public university students in South Africa: A qualitative exploratory study
Source: PLOS Ment Health. 2026 Apr 2;3(4):e0000566. doi: 10.1371/journal.pmen.0000566 (PMC13046107; doi:10.1371/journal.pmen.0000566)
Supplement: S2 Data — (DOCX) [file pmen.0000566.s002.docx]

| **Framework Illustrating Drivers of Psychological Stress Among Black First-Year Female Students from Low-Income Households in a South African Public University** | | | |  |
| --- | --- | --- | --- | --- |
|  | **Academic** | **Financial** | **Social** | **Barriers to utilizing services** |
| **Individual** | Limited skills to navigate academic transition; Limited awareness and access to available academic support | Limited budgeting and personal finance management skills; Fear of dropout due to limited finances | Comparing family background with more affluent peers and feeling “less” | Lack of awareness of services & negative perceptions of services |
| **Peers** | Challenges with group learning dynamics and comparing academic performance with others | Financial and material pressure to keep up with varsity lifestyle, trends and match peers' spending habits | Pressure to give “black tax” like other peers  Pressure to keep up with peers standards of beauty and dress? | Fear of peer stigma |
| **Intimate Relationships** | Challenges balancing relationships & academic; Limited assertiveness communication skills | Transactional relationships and partner sexual entitlement | Low relationship power and partner-controlling behaviours; Low partner commitment and cheating | Denial, fear of confronting personal issues and end relationships |
| **Family** | Fear of failure, disappointing parents and not qualifying for bursaries | Limited financial support & family misperceptions about bursary funds | Expectations for “black tax” & sense of obligation; accusations of neglect | Cultural beliefs and perceptions limit utilisation of services |
| **Institutional** | Limited awareness and access to available academic support, Challenges navigating change from high school to university modes of learning. | Delays in bursary disbursement | Housing challenges and shared living arrangements create a social environment that can facilitate comparison among peers. | Limited visibility and awareness raising around available services. Challenges in scheduling and responding effectively with clients seeking help |
