# Supplementary material for: Drivers of psychological distress among first year female public university students in South Africa: A qualitative exploratory study
Source: PLOS Ment Health. 2026 Apr 2;3(4):e0000566. doi: 10.1371/journal.pmen.0000566 (PMC13046107; doi:10.1371/journal.pmen.0000566)
Supplement: S3 Data — (DOCX) [file pmen.0000566.s003.docx]

**COREQ checklist**

| **Criteria** | **Detailed information** | **Where is the information stated?** |
| --- | --- | --- |
| **Domain 1: research team and reflexivity** |  |  |
| *Personal Characteristics* |  |  |
| 1. Facilitator | The focus group discussions were facilitated by two trained female Black African facilitators (MTM and PM), who are also co-authors. Their shared gender identities with the participants helped to build trust and culturally sensitive engagement during data collection and ensured an inclusive research environment. | Methods Data collection section- Pages 6-7 |
| 1. Researcher credentials and occupation | All authors are affiliated with South African academic and research institutions and have expertise in mixed-methods public health research, with a focus on gender-based violence and mental health. Their combined experience supports rigorous investigation into these critical social and health issues within the South African context. | This checklist & Author affiliation section. |
| 1. Relationship with participants | The facilitators had no prior relationship with the participants, which helped minimize potential bias during data collection. | This checklist |
| 1. Gender | Participants and FGD facilitators were female | This checklist  Methods Data collection section- Pages 6-7 |
| 1. Experience and training | MTM and PM are trained in qualitative research methods and have extensive experience conducting research on gender-based violence (GBV) and mental health with diverse population groups, including women students. Their expertise includes facilitating focus group discussions and engaging sensitively with participants on these topics. | This checklist |
| 6. Relationship established | There was no prior relationship between participants and facilitators before recruitment. Facilitators met the participants for the first time on the day of the focus group discussions and established rapport through introductions and by collaboratively setting ground rules for engagement at the beginning of each session. | This checklist |
| 7. Participant knowledge of  the facilitator | Prior to recruitment, participants had no previous knowledge of or relationship with the facilitators. During recruitment, peer student research assistants provided participants with information sheets detailing the facilitators’ names, institutional affiliations, and the purpose of the study. This ensured that participants were informed about the researchers and the institution conducting the study before participation. | This checklist |
| 8. Facilitator characteristics | FGD facilitators (MTM & PM) are seasoned Black African Female researchers | Methods Data collection section- Pages 6-7 |
| **Domain 2: Study design** |  |  |
| Theoretical Framework |  |  |
| 9.Methodologcal orientation and theory | The study followed a phenomenology approach in qualitative research and social determinants of mental health | Study design Lines 172-173  Background section pages 3-6 |
| Participant Selection |  |  |
| 10. Sampling | Participants were purposively selected based on the following criteria: first-year female students aged 18 to 30 years, recruited from three campuses, who volunteered to participate and provided written consent. | Methods Study design, Recruitment and Data collection section- Pages 6-7 |
| 11. Method of approach | Participants were purposively recruited using multiple strategies, including flyers posted on campus, announcements on institutional social media, invitations circulated via student WhatsApp groups, direct recruitment by a peer research assistant, and referrals from staff in student support services, peer education, and health promotion. Interested first-year female Black-African students aged 18–30 years across three South African university campuses contacted the researchers to participate. Three focus group discussions (15–20 participants each, total n=54) were conducted in isiZulu or English, facilitated by two female Black-African researchers. Sessions lasted 60–90 minutes and were guided by a semi-structured discussion guide to explore psychological distress drivers. | Methods Study design, Recruitment and Data collection section- Pages 6-7 |
| 12. Sample size | We conducted three FGDs with 54 first year female students. FGD1, Campus 1 was attended by 19 participants; FGD2 Campus 2 was attended by 15 participants and FGD3 Campus 3 was attended by 20 participants | Abstract and Line 185 - 186 |
| 13. Non-participation | Four first-year female students from Campus 2 and one from Campus 1 who initially expressed interest in participating were unable to attend the focus group discussions due to scheduling conflicts with practical and other academic activities. | This checklist |
| Setting |  |  |
| 14. Setting of data collection | The FGDs were conducted face to face on 3 campuses which were selected in consultation with the community Advisory Board established for the study | Methods Study design, Lines 172-173 |
| 15. Presence of non-participants | Peer research assistants responsible for participant recruitment also assisted with administrative and logistical tasks, including managing and filing participant consent forms, collecting attendance registers, organizing refreshments, and disbursing reimbursements. | This checklist |
| 16. Description of sample | Participants were Black African, first-year undergraduate female students aged 18–30 years, enrolled at the selected university campuses. |  |
| Data collection |  |  |
| 17. FGD guide | The semi-structured focus group discussion (FGD) guide was developed based on the study objectives. It included open-ended questions designed to elicit participants’ experiences and perceptions regarding the drivers of psychological distress and mental health challenges among first-year female students. | Data collection section- Pages 6-7  Supp-FGD guide excerpt |
| 18. Repeat FGDs | We did not perform any repeated FGDs. | This checklist |
| 19. Audio/visual recording | The FGDs were audio-recorded using a PR200 cellphone recorder, transcribed, and translated into English when conducted in local language. | Data analysis page 7 line 202-203 |
| 20. Field notes | We did not take any field notes. | This checklist |
| 21. Duration | The FGDs ranged in length from 60 and 90 minutes. | Data collection Page 6 Line 188 |
| 22. Data saturation | Probing during the focus group discussions continued until data saturation was achieved, indicated by the point at which no new themes or information emerged from the participants. | This checklist  Methods, Data collection section Page 7 lines 194-197 |
| 23. Transcripts returned | Transcripts of FGDs were returned to participants for correction and confirmation during member checking. | Methods section Data analysis Page 7 Lines 203-216 |
| **Domain 3: analysis and findings** |  |  |
| Data analysis approach: | We used and inductive thematic analysis approach | Methods section Data analysis Page 7 Lines 203-214 |
| 24. Data analysis | The coding team immersed themselves in the transcripts through repeated readings to develop a deep familiarity with the data. Initial manual coding was performed using MS Word, generating data-driven codes aligned with the research questions. The codebook was developed iteratively, with new codes added as they emerged during independent coding. The team then collaboratively grouped similar codes into themes and sub-themes, engaging in continuous discussions to explore their relationships. | Methods section Data analysis Page 7 Lines 203-214 |
| 25. Description of the coding tree | Four main themes emerged as domains driving psychological distress among students: (1)academic transition, (2)social adjustment, (3)financial pressure, and (4) barriers to utilizing available services. Each theme included sub-themes reflecting the students’ individual perceptions of themselves and their interactions with peers, intimate partners, families, and the institutional environment, illustrating how these relationships influenced their experiences of psychological distress. | Results Page 8 section Lines 225-238 |
| 26. Derivation of themes | The key themes were derived from the data. | Methods section Data analysis Page 7 Lines 203-216 |
| 27. Software | We did not use any software; we used MS Word 365 version to manually code the data | Methods section Data analysis Page 7 Lines 203-216 |
| 28. Participant checking | Member checking was conducted with students in each campus who provided feedback on the findings | Methods section Data analysis Page 7 Lines 203-216 |
| Reporting |  |  |
| 29. Quotations presented | Participant quotations were presented verbatim in the results section to illustrate the identified themes and findings. Quotations originally spoken in isiZulu were transcribed and translated into English by research assistants, then reviewed and verified for accuracy by co-investigators fluent in isiZulu. Each quotation was labelled with a non-identifying code (e.g., campus number) to indicate its source while maintaining confidentiality. No participant names or specific campus names were disclosed to protect participant anonymity. | Results section pages 8-18  Ethical considerations Page 7-8 |
| 30. Data and findings consistent | The study findings were firmly grounded in the data, as the thematic analysis accurately reflected participants’ lived experiences related to intersecting identities and the multifaceted challenges of social, financial, and academic adjustment. The themes captured how students perceived these difficulties to be influenced by their relationships with peers, intimate partners, and families, as well as their awareness of and access to institutional support during their transition to higher education. This alignment between the data and findings demonstrates a consistent and credible interpretation of the participants’ perspectives. | Results Pages 1-18  and Discussion Pages 18-21 |
| 31. Clarity of major themes | We described the 4 major themes and the sub-themes in the results section and tabulated them in Supplementary File 1. | Results section Page 8 Lines 227-240 |
| 32. Clarity of minor themes | N/A |  |
