# Supplementary material for: Drivers of psychological distress among first year female public university students in South Africa: A qualitative exploratory study
Source: PLOS Ment Health. 2026 Apr 2;3(4):e0000566. doi: 10.1371/journal.pmen.0000566 (PMC13046107; doi:10.1371/journal.pmen.0000566)
Supplement: S4 Data — (DOCX) [file pmen.0000566.s004.docx]

| **Codebook and Minimal Data** | | | |
| --- | --- | --- | --- |
| **Main Code** | **Sub-code** | **Sub-code 1** | **Example of quote** |
| **Drivers of stress** | **Academic adjustment** | **Adjusting to increased workloads** | “One of the things that make first years have problems, at first is that we try to adjust to schoolwork. It's now big. It's getting out of hand. There are now practicals, and you need to go to campus, and you need to do four modules compared to high school. You used to do eight subjects, but a lot has changed, and you need to adjust to new ways of studying because the way we used to study is no longer the same as what we are doing right now” FGD2, Campus 2 Participant  I think the pressure that we get from school is big. Every Tuesday we have a quiz on that day you have a quiz and then on Thursday you are supposed to go for another assessment. On Friday, where do you get the time to cover for a test that you are supposed to write on Friday. And then you start thinking, "Okay I am going to pass this, I am going to pass this test." Like you stop prioritizing now that okay. So like now "Okay even if I get 50%, that's okay with me." I think that thing causes stress I think another issue is like having to adjust from having to learn a chapter that you learned for two weeks to just two days. I think it's the one thing that... for me that was a biggest challenge because at least if I do not understand the teacher in high school, she will say "Okay, we will try again when we have time later." But now, you understand or not we move on, and you get left behind. And sometimes you get lecturers that you can see they have no time. She is reading the textbook, not that she is adding he knowledge. FGD2, Campus 2 Participant  To add on what she said, here what I have observed is that you have to do your own planning and we were not used to this. You have to do everything at your own time and they do not care how much workload we have, they just keep piling it on. We have two assignments that are due one day after the other. How do you do them? And the practical’s, and quizzes, it's a lot... they don’t care they just give you more work. They make you do 6 chapters. FGD2, Campus 2Participant  The problems pile up. Whilst you are trying to process news about the computer module then you hear others talking about the deadline for the history assignment and an open quiz, everything becomes overwhelming. Then you start to realise that maybe you won’t be able to cope in varsity  FGD 1, Campus 1 Participant |
| **Drivers of stress** | **Academic adjustment** | **Adjusting to new learning methods** | "…I never did accounting in high school, and there are many challenges. You have to learn, and we are not familiar with lecturing. You are used to being taught ‘this, and then that’—but now you are being lectured, and you don’t understand anything. You don’t even know the basics. You see that you are getting lost. You just feel stupid. See, I am stupid." – FGD2, Campus 2 Participant  To add on what she just said, the first speaker, one of the things that make first years have problems, at first is that we try to adjust.It's now big. It's getting out of hand. There are now practicals and you need to go to campus and you need to do four modules but compared to high school you used to do eight subjects but a lot has changed and you need to adjust to new ways of studying because the way we used to study is no longer the same to what we doing right now. FGD3 campus 3 participant  There are many challenges. You have to learn and we are not familiar with lecturing. You are used to being taught that now this and that, but now you are being lectured and you don't understand anything. You don't even know the basics. You see that you are getting lost. You just feel stupid (muncu). See that I am stupid FGD2, Campus 2 Participant  Sometimes for group work, it possible to get selected in a group with “ghost members” (lazy members who disappear and do not contribute to group work ). [Group Laughing]. Ghost members contribute nothing during group work but still expect to receive a mark. Sometimes those type of group members take advantage of the rules set by the lecturer for example if the lecturer said that no group work must be submitted by one person then the lazy members utilize that opportunity by contributing nothing because they know that they won’t get excluded. It’s very common for group work to be done by only three people but at the end everyone receives a mark including those that contributed nothing to the group” FGD3, Campus 3 Participant |
| **Drivers of stress** | **Academic adjustment** | **Less support from lecturers** | Another thing is that the university environment is different from that of a high school one. In high, if you are unable to complete your assignment before the due date, you can talk to the teacher who gave you an assignment and explain your situation then ask for an assignment due date to be extended, whereas in varsity if you don’t submit your assignment on time then the system locks you out. And it’s very rare for a lecturer to be lenient and extend the due date for an assignment. Sometimes for group work, it possible to get selected in a group with “ghost members” (lazy members).  [Group Laughing] [Group Laughing]” FGD3, Campus 3 Participant  There are many challenges. You have to learn and we are not familiar with lecturing. You are used to being taught that now this and that, but now you are being lectured and you don't understand anything. You don't even know the basics. You see that you are getting lost. You just feel stupid (muncu). See that I am stupid – FGD2, Campus 2 Participant |
| **Drivers of stress** | **Academic adjustment** | **Limited information provided about academic support** | “The problem is that we don’t know where the offices for the lecturers are located, and some lecturers want you to set an appointment before coming to their offices. It’s a long process. We are not yet familiar with a lot of buildings on campus, and we only know a few offices where we can find our lecturers. Having no idea where we can find most of our lecturers is a big issue.” FGD3, Campus 3 Participant |
| **Drivers of stress** | **Academic adjustment** | **Fear of failure and losing access to bursary funding** | “I completed a certain module a week before we closed. I didn't know that was my last test and we are writing on campus. Like everything got compacted into two tests. Like, how can you do that? How am I going to cover all these topics? And you are supposed to get As. You are supposed to get As to maintain the bursary. I mean okay at least there is like counselling here on campus, but yoh, I was left traumatised”. FGD2, Campus 2 Participant  Sometimes you know this thing of maybe you do well in matric, and then you come to varsity, you actually... you don't go from... maybe academics drop to like 80s, sometimes you just drop to a 30 and then you can't understand how you can get that and then you see other kids doing well and you think "What's wrong with me?" Sometimes you even question like, "Was I really that smart or I was just wasting my time?" FGD2, Campus 2 Participant  I think it’s the pressure of being in varsity, there is this pressure to excel in academics especially if you were one of the top achievers in high school. Former high school teachers also add unnecessary pressure by asking you if you are still performing well in varsity as you did in high school. When one starts failing in varsity, one realizes that life is difficult, it gets worse when your parents put pressure on you by having high expectations of you in varsity particularly in academics. FGD1, Campus 1 Participant  The other thing is people we stay with and those we meet when you arrive here, you are a first-year student. The person you meet and you tell them which course you are doing, like she will exclaim like, "Hawu, you are doing such a difficult course. Do you know there are only seven postgrad students in that course?” Then you think, "How am I going to survive in this course?", because when I arrived here, I was sitting in the library, I was studying because I was writing the next day. Then people came, "Hawu you are sitting here you are writing such a difficult module. Forget it you are not going to pass… You must use the guideline." I don’t even know what that guideline is. Where is that guideline? And I was so frustrated that, okay there is nothing I can do then. FGD1, Campus 1 Participant  And sometimes they just say, "What are you studying?" and you say, "I'm doing this?" Then they say, "Yoh, where are you going to get a job?" FGD1, Campus 1 Participant |
| **Drivers of stress** | **Academic adjustment** | **Less emotional support from family & pressure to succeed** | I think it’s the pressure of being in varsity, there is this pressure to excel in academics especially if you were one of the top achievers in high school. Former high school teachers also add unnecessary pressure by asking you if you are still performing well in varsity as you did in high school. When one starts failing in varsity, one realizes that life is difficult, it gets worse when your parents put pressure on you by having high expectations of you in varsity particularly in academics. FGD1, Campus 1 Participant  Parents also put us under a lot of pressure regarding our schoolwork and provide less support to us. There is a student that I know, she was stressed about school and her parents didn’t take her serious until she attempted to commit suicide. FGD1, Campus 1 Participant |
| **Drivers of stress** | **Life adjustment** | **Challenges with accommodation** | You see, kids who come from places that are far face greater challenges. If you are from near or have family, you can at least phone and say, 'Please help me.' But if you are coming from far, you are stuck here at the university with nowhere to turn because you do not have family in the province." FGD2, Campus 2 Participant  “Especially if you are coming from another province you might end up having to go back without getting residence which is disadvantageous and need to be taken care of. Unfortunately, with this issue of residences, I think institutions are not determined to fix residence challenges hence it been years experiencing the same issues.” FGD3, Campus 3 Participant |
| **Drivers of stress** | **Life adjustment** | **Challenges with adulting and becoming independent of families** | P: In public schools, learners are forced to abide by the rules of the school like wearing school uniform and there are women which are hired to cook for learners, so the learners are not taught how to be independent.  P: So, you have to ‘parent’ yourself.  Facilitator: So, you have to ‘parent’ yourself as well?  P: Yes  Facilitator: ‘parenting’ yourself is also stressful?  P: Yes.  Facilitator: Why is it stressful? Because having freedom means you can do anything you want.  [Everyone talking at the same time]  P: You have to parent yourself. That is where the problem starts. Having no one to provide you with guidance. FGD1, Campus 1 Participants  So firstly to adjust from knowing that the parents were protecting you to now having the freedom which sometimes if you don’t have set morals and values you end up agreeing to do anything; then later on you start regretting, you then realise that, "Actually I shouldn't have or I wasn't supposed to go there or I wasn't supposed to do this and that." So I think that is just one of the issues FGD1, Campus 1 Participant  P: When you are doing groceries for your home, food prices don’t really matter unlike when you are doing your own grocery, the price of every item that you purchase matters the most.  P: It’s worse when you must leave some important items in the store due to a tight budget, so that is also challenging FGD1, Campus 1 Participants |
| **Drivers of stress** | **Financial stressors** | **Limited financial support from family** | If the university doesn't have funding, then you must ask for everything from your parents. When other students have funding, this ends up affecting your mental health when they are happy that their money has come through and you do not have anything”. FGD2, Campus 2 Participant  Some parents provide financial support to their kids in varsity, but some delays do occur regarding that money from parents. In a worst-case scenario, sometimes our parents promise to give us money but end up not doing it and don’t even provide any explanation for breaking their promise FGD1, Campus 1 Participant  “Another thing which I think happens to most of us is that when we ask for some money from our parents, they reprimand and accuse us of being careless with the way we handle our finances in varsity. Hearing my parents complaining about money problems at home instead of assisting me financially increases my stress levels.” FGD3, Campus 3 Participant  Our parents have this assumption that just because we have funding then it means that there is no need for them to support us financially. Our parents think that R1500 is enough to sustain us for the whole month. Sometimes we often feel pressured to buy unnecessary things that exceed our budget if we notice that other students are buying a lot of things FGD1 Campus 1 Participant |
| **Drivers of stress** | **Financial stressors** | **Family demands to send some of their bursary monies (NSFAS)** | “I think the other stressful thing is black tax. Sometimes, even though one will not agree, back home they expect her to give them R500 from the money she gets from NSFAS or bursary to help her siblings because some of them, you find she is the first generation that will graduate from her family. So, if the parent maybe doesn't have a good job and then you have to make a plan”. FGD2, Campus 2 Participant  “I don’t mean this in a bad way but some of our parents think that when we are here and getting NSFAS, it's like we are getting a lot of money. They raised and sent us to school from nothing. They now think that we must help them because there is money that we are getting. So now when you are saying you cannot help your parents, they think that you have forgotten them back home. You are now living this life. "Every time we ask for help, you say you don’t have one, but there are things you need from us. You don’t care about us” FGD2, Campus 2 Participant  “Firstly is that situation you are going through where you find that your mother, maybe she is not working. The minute you do not send money, you will feel guilty because you know the situation you left at home. Maybe you have to sacrifice some money because you will think why can’t I also do like others and give them money too.” FGD2, Campus 2 Participant  Having no NSFAS funding causes a lot of stress. You will be overthinking about dropping out of school because having no funding causes a lot of stress.” FGD3, Campus 3 Participant  “I think the other stressful thing is black tax. Sometimes, even though one will not agree, back home they expect her to give them R500 from the money she gets from NSFAS or bursary to help her siblings because some of them, you find she is the first generation that will graduate from her family. So, if the parent maybe doesn't have a good job and then you have to make a plan”. FGD2, Campus 2 Participant  P: Yes, another thing is that if you know that you come from a poor background, whenever you get your allowance you feel like you must send some money home to meet them halfway.  Facilitator: So, they are expecting..?  P: Yes they are expecting. If you have NSFAS funding they know you get monthly allowance therefore expect money from you. FGD1, Campus 1 Participants  “But you know the situation at home. We are living from pay check to pay check. So why are you demanding these things to impress your friends at the expense of your parent?" Sometimes the siblings in general they will call and say, "My money is finished. Please top me up", then the parent will take from the money that maybe she was going to pay for electricity and something else, and now there is shortage of money maybe for petrol R300 per month. And then sometimes they use this line, "No, I'll pay you back on the 15th." You end up giving them and saying, "No, there is no problem. FGD1, Campus 1 Participant |
| **Drivers of stress** | **Financial stressors** | **Challenges in managing personal finances** | “You have to ‘parent’ yourself. That is where the problem starts. Having no one to provide you with guidance.” FGD1, Campus 1 Participant.  Sometimes you have to sacrifice food to buy clothes,” and “When you are doing your own grocery, the price of every item matters the most.” FGD1, Campus 1 Participant.  “Once the allowance starts reflecting on our accounts, we begin to become stressed about how we are going to use R1500 (USD 80) to sustain us throughout the month.” FGD3, Campus 3 Participant  NSFAS allowances are unstable. Prior to receiving allowances, we sometimes find ourselves drowning in debts. There was this one time when I ordered an expensive perfume, UKZN T-shirt and a cap before receiving my monthly allowance. So, after I had received my allowance, I had to pay for those things that I ordered, and it wasn’t my best moment. The following day I had to buy grocery for R500, unfortunately due to high food prices I ended up exceeding my budget. FGD 2 Campus 2 Participant  On top of that, we as women like to smell good, so we usually order perfumes prior receiving NSFAS allowances then pay as soon as we receive our allowances. But it’s not usually a nice moment because the people that sells us perfumes remind us to settle our debt as soon as we receive the monthly allowances. If the cost of the perfume was R300 then it means that the other R300 will be remaining. There are also temptations such as seeing other students ordering food online. FGD2, Campus 2 Participant |
| **Drivers of stress** | **Financial stressors** | **Transactional relationships** | “If ever there is someone you know who is dating a blesser or doing whatsoever, please do not judge those people. Try to understand why they did these things in the first place because you find that some of them are expected to support their families back home. One may have left a home that does not have anything at all and then she will have the pressure of trying to maintain standards. It's a lot”. FGD2, Campus 2 Participant  “Maybe you have found yourself a blesser. Now you have to balance when the blesser needs you, you have to be there and your books are there too and you must pass, because when you don’t go to the blesser, then you will not get anything. You won’t get money; you won't get anything if he doesn't get you.” FGD2, Campus 2 Participant  I wanted to add on the point of money. Some females end up deciding to get sugar daddies so they can get extra money. This decision can backfire hence the sugar daddy who is giving you money might end up controlling you resulting to more stress. FGD1, Campus 1 Participant |
| **Drivers of stress** | **Social adjustment** | **Peer pressure to keep up with lifestyle trends** | “Seeing other students going out to celebrate receiving their allowances makes me more stressed... I feel pressured to go out too, though I know I must use that money wisely.” FGD3, Campus 3 Participant  “When going with friends they expect you to go and if you tell them, you don’t not have money, they will not understand hence they do not relate to an unfortunate background. Sometimes we often feel pressured to buy unnecessary things that exceed our budget if we notice that other students are buying a lot of things”. FGD3, Campus 3 Participant  P: I will speak on behalf of people which have funding. It easy for people who are not in varsity to judge us as students especially when it comes to buying clothes and the way we dress as students. Clothes is a “need” …  Facilitator: when you say “need…  P: Clothes are an essential aspect when you are in varsity. Some students do a budget when they receive an NSFAS allowance for instance R800 will be used to buy clothes then the remaining money will be used to buy food. The problem arises when the remaining money from the allowance becomes insufficient to buy enough food for the whole month hence we run out of food, sometimes  FGD1, Campus 1 Participants  P: "Firstly, one needs to dress nice… and doing shopping requires a lot of money. One has to dress nice without repeating clothes to appear attractive to others." [Group laughing]  P: "One might make silly comments if you are always wearing the same hat every day."  P: "And that might make you feel even ashamed to ride a school bus." FGD1, Campus 1 Participants  Sometimes we stay with postgraduates and some of us are trying to reach the standards and then end up being introduced to some certain situations whereby you have to do stuff to... well to maintain that standard that you cannot reach. Then at some point you end up regretting and getting into stuff that you never thought you will ever be in sometimes." FGD2, Campus 2 Participant.  "Maybe you might sometime stay with a roommate who is, maybe her background, comes from a family that is financially stable and then at some point she will do stuff that will make you have an interest in them... Then you start learning new ways of making extra income." FGD2, Campus 2 Participant  "Sometimes the dress code creates pressure because some people on campus look at you in ways that make you feel uncomfortable or insecure. This leads you to try to dress like them, even when you cannot afford it, because the money you have only covers your basic needs.” FGD2, Campus 2 Participant |
| **Drivers of stress** | **Navigating intimate relationships** | **Controlling behaviours** | “Another thing that I want to add is that sometimes, isn't when we arrive as first years, it is rare for you to date another first year. So, like when you are dating seniors, they are always calling you and they isolate you from your friends so that whatever he says to you, you will feel like it's the right thing.” FGD2, Campus 2 Participant.  “Sometimes guys like to dictate how their girlfriends should dress and whom they should hang out with. If a girl doesn’t follow those rules, then hell breaks loose.” FGD1, Campus 1 Participant  “Those with boyfriends that they cook for at residences. Some boys do not understand how busy you will get. You have told him how busy you will be, like "So you will not have time for me." And other boyfriends who are not studying get cooked for here. A person will say, "What have you cooked there mama? If you are dating someone who is not studying when you are studying, you find that you have schoolwork, and he won’t understand that today I can’t show up because it's a lot. Like I have to study. He expects you to be there when he wants you to be there. That's just not it.” FGD2, Campus 2 Participant  “Knowing that my boyfriend doesn't trust me and keeps on dictating how I should live my life and whom to hang out with increases my stress level. The worst part is when you can’t even have a simple conversation with your boyfriend because of how he reacts to a lot of thing”. |
|  |  | **Abuse, cheating and hurt in relationships** | “You find that other people get violated within relationships. A person will say, "No I have spent this money on you. Now when we must do this you are telling me what to do, it happened a long time ago. So why can’t you move on? Things like that.” FGD2, Campus 2 Participant  " Sometimes as female students we don’t get along due to dating a same guy at the same time. So, sometimes things can get overwhelming when you are dealing with academic stress, and pressure from your peers telling you to remain strong and keep on dating a guy who is cheating on you. It possible as a female student to fail a module because you couldn’t ask for assistance from a girl you are sharing a man with.Males in varsity are never serious when it comes to romantic relationships, it's like they plan to deliberately hurt girls. In my own experience, I've been in a couple of relationships ever since I arrived in varsity, but it was not a serious thing. What I noticed is that female students who are in serious relationships with comrades get hurt the most because they get played. Dating a male comrade is a worst thing one can ever do. During razzmatazz, you hear some students gossiping about the comrades’ saying things like “did you see that girl dancing too close with a particular male comrade  [Group laughing] FGD3, Campus 3 Participant  P: “In my own experience, I’ve been in a couple of relationships ever since I arrived in varsity, but it was not a serious thing. What I noticed is that female students who are in serious relationships with comrades get hurt the most because they get played.  Facilitator: They get played---  P: They get played. Sometimes a guy may be dating three girls at once without hiding it. It's worse for a girl who already has strong feelings for that cheating guy.” FGD3, Campus 3 Participant.  “A guy that is not cheating on you becomes too much… he becomes overprotective of you and obsessed.” FGD1, Campus 1 Participant |
| **Mental health helpseeking** | **Barriers to utilizing campus mental health support services** | **Booking challenges & other access issues** | "The support systems here at varsity I tried to apply, it kept on repeating the same thing to me. You can’t apply. You apply and they say it's fully booked, fully booked and I ended up giving up." FGD2, Campus 2 Participant  P: What I can say is, I don’t want to lie. Like here at UKZN there are support structures but at the same time it's just (pause)....  Facilitator: It's tough  P: Not that it's tough but it's a thing of like, it's not proper support but then they claim that there are support structures but it's not proper. For example, if you try and book a session with a therapist that is here, it's either it's fully booked and you have to wait two weeks, so you are sitting with this problem for two weeks. There is nothing you can do.  FGD2, Campus 2 Participant |
| **Mental health helpseeking** | **Barriers to utilizing campus mental health support services** | **Limited information & uptake** | P:"We lack information about these services. If I were to start searching for these services, I’m sure that it would take me a lot of days to find them."  P: "Another thing is that we don’t utilize those services on campus because their entrances are hidden... it’s difficult to locate venues for psychologists. Maybe it would have been better if there were big boards with signs directing us to the location of the psychologists." FGD1, Campus 1 Participants  P: There are services which are available on campus to assist with those issues, even the mentorship groups also provide information on who to contact to get assistance about those issues. The only problem is that we as individuals don’t bother ourselves to ask for that kind of assistance  FGD1, Campus 1 Participant |
| **Mental health helpseeking** | **Barriers to utilizing campus mental health support services** | **Service perceptions** | “I once attended counselling here... but it didn’t feel structured. I was put in a space with too much freedom, where I was expected to come up with my own solutions without clear advice or options laid out. I needed someone to guide me through possible steps, not just listen broadly." FGD2, Campus 2 Participant  P: The thing is, people are not the same. I’ll make an example about me, I have this belief that whenever I feel stressed or overwhelmed by problems, opening to someone else about those problems isn’t a good idea so I just bottle things up. Though services that offers counselling are available on campus, but we are scared to utilize those services and open up. Sometimes, we fear being judged…  Facilitator: You fear being judged…  P: Yes. From my experience, there is someone I know who once attended those counselling sessions on campus. She found out that counselling sessions on campus are like focus group discussions where everyone sit in a group and share their problems. The disadvantage of a counselling session like that one is that it makes one feel exposed. As a result, one end up not attending any more of those sessions.  FGD3, Campus 3 Participant |
| **Mental health help seeking** | **Barriers to utilizing campus mental health support services** | **Fear of mental health stigma** | P:“It’s time, we are always busy with schoolwork.  P: The type of friends that we have also prevents us from seeking help from those services. So, to avoid being labeled as a ‘lunatic’…" FGD1, Campus 1 Participants |
| **Mental health help seeking** | **Barriers to utilizing campus mental health support services** | **Challenges on following through actions after accessing services** | "I think that one of the reasons we don’t attend counselling is because we fear dealing with the truth." FGD3, Campus 3 Participant.  "For example, if you talk to the psychologist about a toxic boyfriend then obviously the psychologist will advise you to dump your boyfriend which is something that one doesn’t want to admit. So, we really don’t want to face the truth."FGD3, Campus 3 Participant |
